# Geographic Structuring and Divergence Time Frame of Monkeypox Virus in the Endemic Region

**DOI:** 10.1093/infdis/jiac298

**Published:** 2022-07-14

**Authors:** Diego Forni, Cristian Molteni, Rachele Cagliani, Manuela Sironi

**Affiliations:** Bioinformatics, Scientific Institute IRCCS E. MEDEA, Bosisio Parini, Italy; Bioinformatics, Scientific Institute IRCCS E. MEDEA, Bosisio Parini, Italy; Bioinformatics, Scientific Institute IRCCS E. MEDEA, Bosisio Parini, Italy; Bioinformatics, Scientific Institute IRCCS E. MEDEA, Bosisio Parini, Italy

**Keywords:** molecular dating, monkeypox virus, *Orthopoxvirus*, population structure

## Abstract

**Background:**

Monkeypox is an emerging zoonosis endemic to Central and West Africa. Monkeypox virus (MPXV) is genetically structured in 2 major clades (clades 1 and 2/3), but its evolution is poorly explored.

**Methods:**

We retrieved MPXV genomes from public repositories and we analyzed geographic patterns using STRUCTURE. Molecular dating was performed using a using a Bayesian approach.

**Results:**

We show that the population transmitted in West Africa (clades 2/3) experienced limited drift. Conversely, clade 1 (transmitted in the Congo Basin) possibly underwent a bottleneck or founder effect. Depending on the model used, we estimated that the 2 clades separated ∼560–860 (highest posterior density: 450–960) years ago, a period characterized by expansions and contractions of rainforest areas, possibly creating the ecological conditions for the MPXV reservoir(s) to migrate. In the Congo Basin, MPXV diversity is characterized by 4 subpopulations that show no geographic structuring. Conversely, clades 2/3 are spatially structured with 2 populations located West and East of the Dahomey Gap.

**Conclusions:**

The distinct histories of the 2 clades may derive from differences in MPXV ecology in West and Central Africa.

## Teaser

Within the endemic region, MPXV clades show distinctive geographic structuring of genetic diversity. Separation of major lineages occurred in a period ranging from the Medieval Warm Period to the Little Ice Age, which was characterized by changes in rainforest coverage.

Monkeypox was first recognized as a human disease in 1970, in the Democratic Republic of the Congo ([DRC] at the time known as Zaire) (https://www.who.int/news-room/fact-sheets/detail/monkeypox). Although it was once considered a rare zoonotic disease confined to West and Central Africa, the incidence of monkeypox has been increasing in recent years, both in humans and in primate communities [1–4]. In the past few years, monkeypox re-emerged in Nigeria and in the DRC, causing large outbreaks [4–6]. In 2018, 4 individuals traveling from Nigeria to the United Kingdom, Israel, and Singapore became the first human monkeypox cases exported from Africa [7]. Previous occurrences of monkeypox outside the endemic region were instead caused by exotic pets imported from Ghana into the United States, in 2003 [7]. Although all of these exported cases generated no or few secondary transmissions, starting from May 2022, an unprecedented monkeypox outbreak is expanding worldwide [8]. As of June 22, 2022, 3413 cases have been confirmed (https://www.who.int/emergencies/disease-outbreak-news/item/2022-DON396), and most of these are thought to have been derived from human-to-human transmissions [9].

No specific treatment or vaccine is available for monkeypox, although smallpox vaccination confers substantial cross-protection [7]. In fact, the causative agent of monkeypox, monkeypox virus (MPXV), belongs to the *Orthopoxvirus* genus (family *Poxviridae*), which also includes variola virus ([VARV] the cause of smallpox) and vaccinia virus (used as the smallpox vaccine). Monkeypox and smallpox have similar clinical presentation, characterized by a systemic disease with vesiculopustular rash [7]. However, unlike VARV, MPXV is a zoonotic virus. Although its animal reservoir(s) is presently unknown, field surveys and analysis of museum specimens indicated rodents as the most likely natural hosts of MPXV [10, 11]. The epidemiology of the disease in the endemic region suggests that the viral reservoir is associated with the African rainforest, although the 2017 Nigerian outbreak also included cases in savanna regions and in urban areas [4].

Like all poxviruses, MPXV has a long (∼190 kb) and complex double-stranded deoxyribonucleic acid (DNA) genome. Phylogenetic analyses indicated geographic structuring of viral diversity with 2 major clades. These were originally named after the regions where the virus is mainly transmitted: West Africa (WA) and the Congo Basin (CB) [12, 13]. However, it was recently proposed that a nondiscriminatory nomenclature for MPXV clades should be adopted (https://virological.org/t/urgent-need-for-a-non-discriminatory-and-non-stigmatizing-nomenclature-for-monkeypox-virus/853). Accordingly, we will refer to MPXV clades as clade 1 (mainly transmitted in the CB) and clades 2 and 3 (mainly transmitted in WA). Viruses causing the current worldwide outbreak belong to clade 3 [9], and the genetic diversity of MPXV might have an effect on clinical features, with suggested higher mortality for clade 1 viruses [7, 13]. Other than this, little is known about the evolutionary history of MPXV, and substantial uncertainties remain concerning the timing and location of viral diversification in Africa [12, 14]. Likewise, a detailed analysis of the spatial structuring of MPXV populations in the endemic region is missing.

## METHODS

### Sequences, Alignments, Network, and Nucleotide Diversity

Complete MPXV genomes were retrieved from the National Center for Biotechnology Information (NCBI) virus database (https://www.ncbi.nlm.nih.gov/labs/virus/vssi/#/) (Supplementary Table 1). Additional details are available in the Supplementary Methods. The scaled number of segregating sites (θ_W_ [15]), the average number of pairwise differences (π [16]), and Tajima’s D statistic [17] were calculated using the POP-GENOME R package [18].

### Linkage Disequilibrium and Population Structure

Population structure analysis was performed by considering biallelic parsimony-informative sites. Additional details are available in the Supplementary Methods. To evaluate linkage disequilibrium (LD) we used the LIAN software (version 3.7) [19].

The STRUCTURE (version 2.3.4) [20] software was applied to evaluate viral population structure. We first estimated the λ (allele frequency spectrum) [21]. Using the obtained λ value of 1.24, the linkage model with correlated allele frequencies was applied [21] for K from 1 to 12 and the optimal K was selected [21, 22]. Because sampling is uneven in terms of geographic representation in both clade 1 and clades 2/3, we ran the analyses using an ancestry prior that allows source populations to contribute deferentially to the pooled sample of individuals (POPALPHAS = 1). This prior allows accurate inferences when sampling is unbalanced [23]. We also ran the analyses using POPALPHAS = 0, and similar results were obtained (data not shown), suggesting that unequal sampling has no major effects on STRUCTURE results.

The amount of drift that each subpopulation experienced from ancestral frequencies was quantified by the F parameter (calculated for the optimal K value) [21]. Additional details on STRUCTURE analyses and LD are available in the Supplementary Methods.

### Recombination Analysis

Recombination analysis was performed using the 3SEQ Recombination Detection Algorithm (version 1.7) [24] with default parameters, with the exception of the P value cutoff. We accepted events with a corrected P < .01 (-t parameter). Breakpoints were mapped on the whole genome alignment based on the 3SEQ output, and the largest nonrecombinant region (ie, the region between 2 breakpoint events) was selected.

### Molecular Dating

To evaluate whether the nonrecombinant genomic region carried sufficient temporal signal, we calculated the correlation coefficients (r) of regressions of root-to-tip genetic distances against sequence sampling dates [25]. Cowpox virus (NC_003663) was used as the outgroup.

We performed a time estimate phylogenetic reconstruction using a Bayesian approach implemented in the Bayesian Evolutionary Analysis by Sampling Trees (BEAST, version 1.10.4) software [26]. Analyses were performed using a strict clock model, a constant population size tree prior, and the HKY85 substitution model, as previously suggested [1, 12].

To account for the time-dependent rate phenomenon (TDRP) [27] in the estimation of the diversification of the MPXV phylogeny, we applied a recently proposed method: the prisoner of war (PoW) model [28]. Additional details on molecular dating and application of the PoW model are available in the Supplementary Methods.

## RESULTS

### Genetic Diversity of Monkeypox Virus Major Clades

We obtained all available complete or almost complete MPXV genomes from public databases and literature sources [1, 4, 12–14, 29–37] (Supplementary Table 1). Sequences with known geographic origin (n = 90) were aligned. Sequences from the ongoing monkeypox outbreak were not included because their ultimate geographic origin is unknown and because selective pressures distinct from those present in the endemic region were suggested to drive their evolution [38].

**Figure 1.**
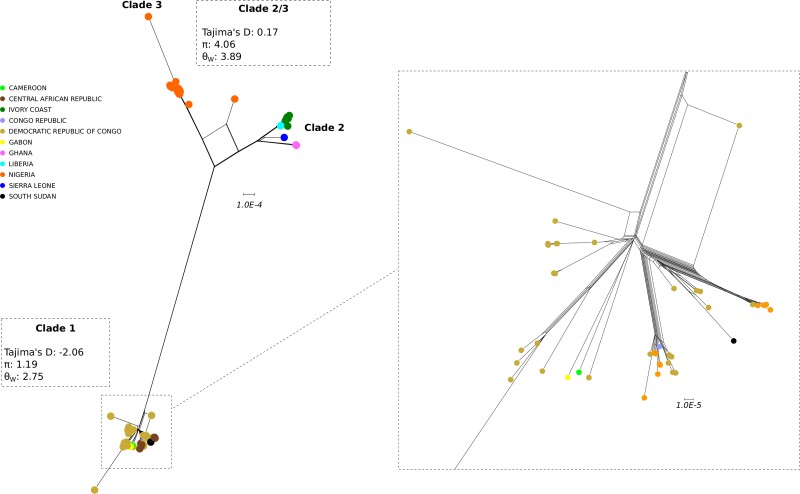
Divergence of monkeypox virus (MPXV) genomes. Neighbor-net split network of 90 MPXV genome sequences. Each sample is shown as a dot and colors represent country of origin (as indicated in the legend). Nucleotide diversity is reported for clades 1 and 2/3, along with an enlargement of clade 1 genetic relationships.

Using the 90 complete genomes, we generated a neighbor-net split network. In line with previous works [12, 13], 2 major clusters of sequences were clearly evident in the network and corresponded to clades 1 and 2/3 (Figure 1). Genetic diversity was higher in clades 2/3, with evidence of geographic clustering roughly corresponding to Nigerian sequences (clade 3) and to genomes sampled west of Nigeria (ie, in the Ivory Coast, Sierra Leone, Ghana, and Liberia) (clade 2).

To gain insight into the demographic histories of MPXV clades, we estimated nucleotide diversity (θ_W_ and π) and Tajima’s D [15–17]. As expected, nucleotide diversity was higher for clades 2/3 than for clade 1 (Figure 1). The former displayed a value of Tajima’s D close to 0, whereas clade 1 had a negative value. D values close to zero are expected in neutrally evolving populations of constant size, whereas negative Tajima’s D values indicate an excess of low frequency polymorphism caused either by background selection, selective sweeps, or population expansion. Although the effects of demography and selection are difficult to disentangle, selective events usually affect a relatively small fraction of sites. Thus, genome-wide estimates are more likely to be indicative of demographic effects. Indeed, a sliding window analysis along the genome, despite fluctuations for both populations, indicated a general trend for clade 1 and 2/3 sequences to have *D* values lower than 0 and approximately 0, respectively (Supplementary Figure 1).

Together with the little diversity observed for clade 1, the value of *D* suggests that this population has experienced population expansion after a bottleneck or a founder effect/migration event. Negative values of *D* (consistent with population size expansion) were instead not observed for clades 2/3.

### Population Structure of Monkeypox Virus in the Endemic Region

We next investigated MPXV population structure and geographic diversity with STRUCTURE [20]. This program relies on a Bayesian statistical model for clustering genotypes into populations without prior information on their genetic relatedness or geographic origin. STRUCTURE can identify distinct clusters or ancestral subpopulations that account for the ancestry of individuals in the overall, extant population. Because STRUCTURE is ideally suited for weakly linked markers [20, 21], we first analyzed the level of LD with LIAN version 3.7 [19]. Statistically significant LD was detected, with a standardized index of association (*I*_A_S) of 0.34, a value indicating moderate LD. The frequency of MPXV recombination in natural settings is presently unknown, but our data (see below) suggest that it is not particularly high. Thus, mutation and variable selective pressures across the genome are likely to contribute to LD. In any case, this level of LD warrants the application of STRUCTURE models. Specifically, we used the linkage model with correlated allele frequencies, which assumes that discrete genome “chunks” are inherited from K ancestral populations [21].

**Figure 2.**
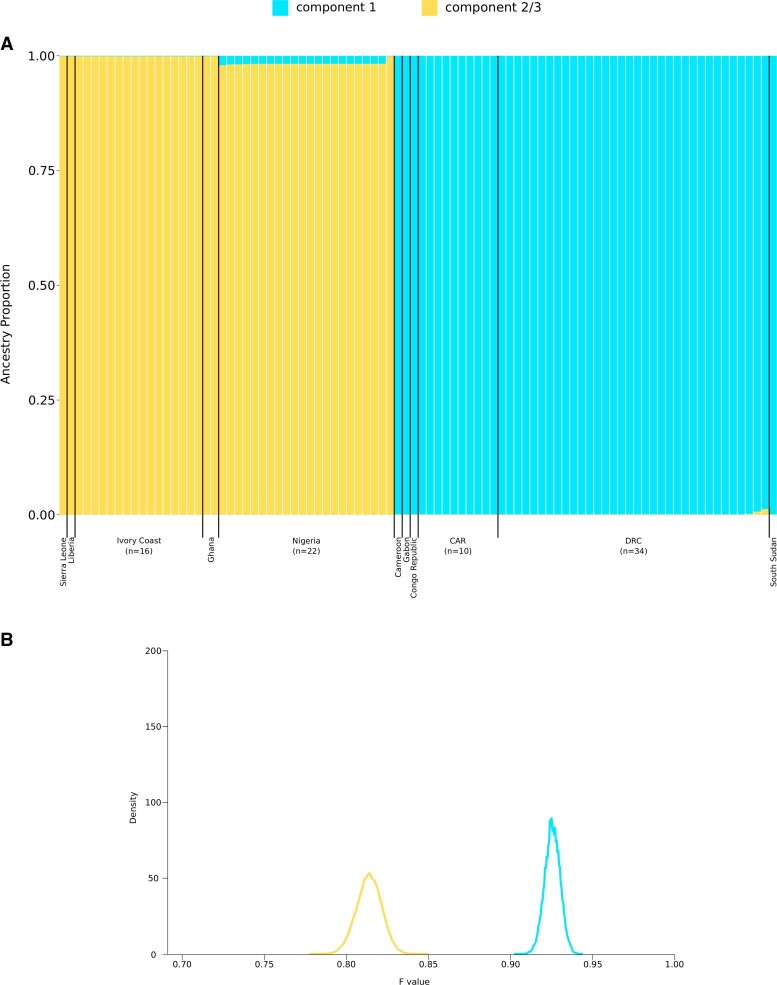
Population structure of monkeypox virus (MPXV). (*A*) Bar plot representing the proportion of ancestral population components for K = 2. Each vertical line represents an MPXV genome, and it is colored by the proportion of sites that have been assigned to the 2 populations by STRUCTURE. Viral genomes are ordered by country (*B*) Distributions of F values for the 2 populations. Colors are as in (*A*). CAR, Central African Republic; DRC, Democratic Republic of the Congo.

The optimal number of populations (K) was estimated using the ΔK method [22] and resulted equal to 2 (Supplementary Figure 2). The 2 ancestry components identified by STRUCTURE clearly correspond to clades 1 and 2/3, with virtually no evidence of admixture (Figure 2*A*).

To gain further insight into the evolutionary history of the 2 populations, we used the linkage model to estimate the level of drift of each subpopulation from a hypothetical common ancestral population. Specifically, we estimated the F parameter, which represents a measure of genetic differentiation between populations based on allele frequencies. The F value for the clade 2/3 component was lower than that of the clade 1 component (Figure 2*B*), indicating that genomes in clades 2/3 diverged less than those in clade 1 from the ancestral common population. This is consistent with, but not proof of, a West African origin of all circulating MPXV strains. This also suggests that the clade 1 viral population experienced a bottleneck or founder effect.

The strong genetic differentiation between the clade 1 and clades 2/3 populations may mask further sublevel clustering. Thus, STRUCTURE analysis was repeated for the 2 clades separately.

**Figure 3.**
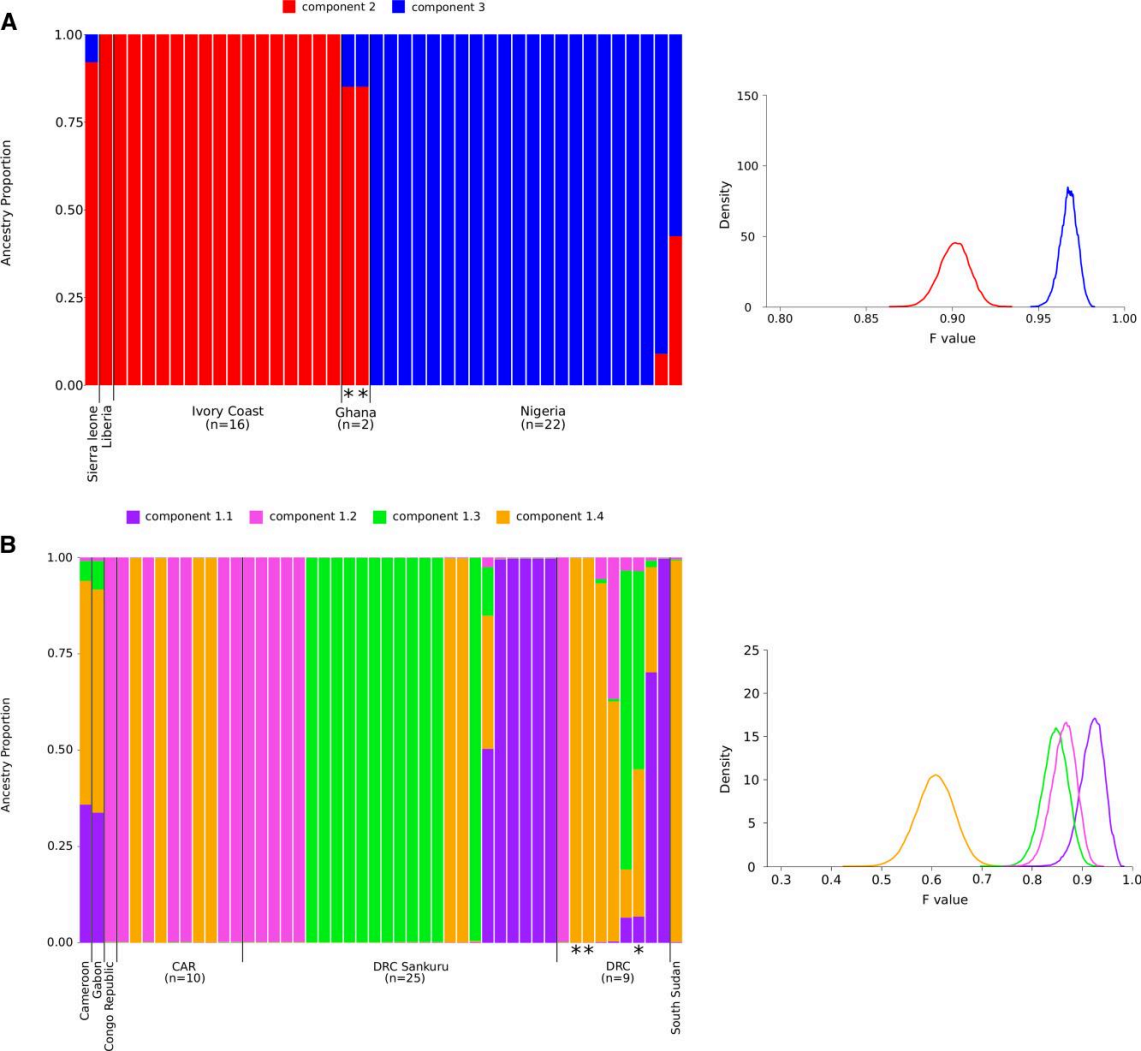
Population structure of monkeypox virus (MPXV) clades. Bar plots representing the proportion of ancestral population components for clades 2/3 (K = 2) (*A*) and clade 1 (K = 4) (*B*). Each vertical line represents an MPXV genome and is colored by the proportion of sites that have been assigned to the different subpopulations by STRUCTURE. Viral genomes are ordered by country. Distributions of F values are also reported next to the bar plots. Asterisks indicate rodent-derived viral samples cited in the text. CAR, Central African Republic; DRC, Democratic Republic of the Congo.

**Figure 4.**
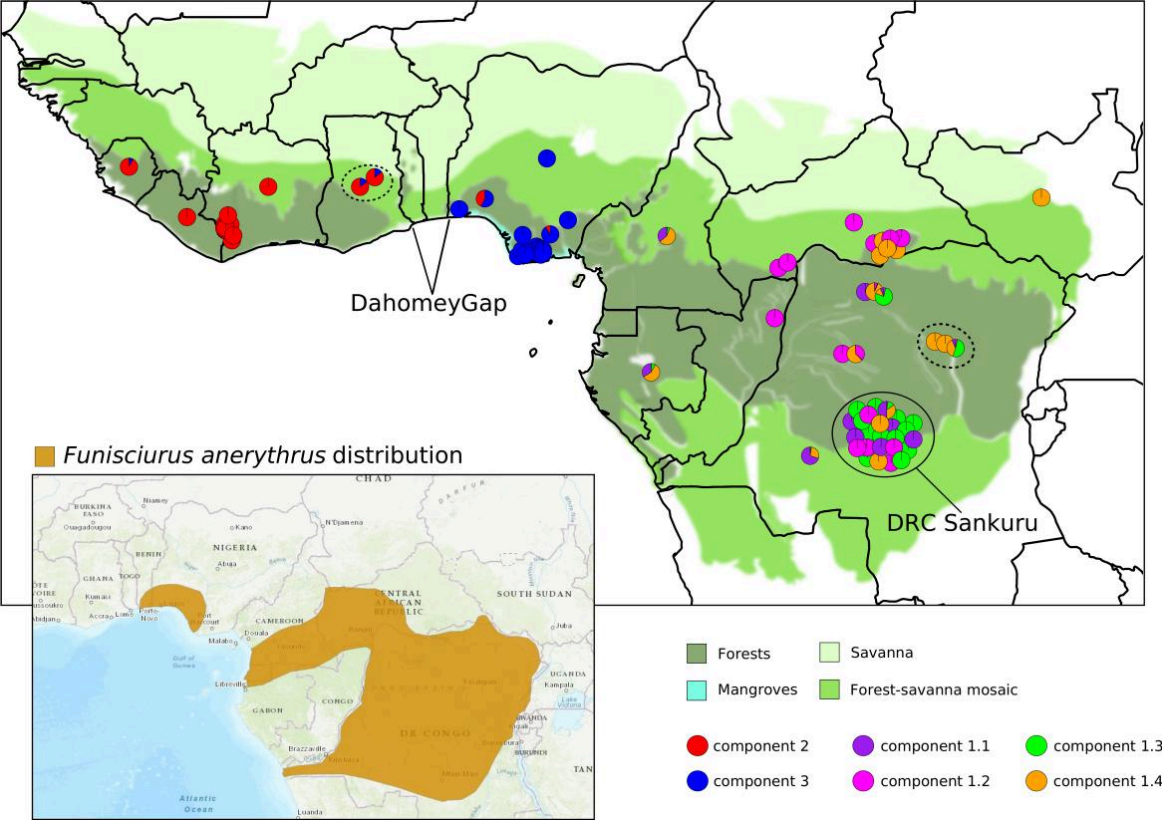
Geographical distribution of monkeypox virus genomes. Each viral sequence is shown as a pie chart, with colors representing the proportion of ancestral population components estimated by STRUCTURE (see Figure 3 and the legend for color scheming). Viral strains are placed on the map using country or, when possible, subregion sampling information (Supplementary Table 1). Identical locations were jittered to allow the visualization of ancestral population components. When “country” was the only available information, strains were placed in the center of that country. Three Nigerian strains were not plotted on the map due to the lack of clear sampling information. Dotted circles indicate rodent-derived viral samples cited in the text. Terrestrial biomes of the sampling locations of viral strains are also shown on the map (see the legend for details). Information was retrieved from the Digital Observatory for Protected Areas (https://dopa.jrc.ec.europa.eu/dopa/). An insert with the geographic distribution of Funisciurus anerythrus is also reported (figure adapted from https://www.iucnredlist.org/). The map was created using maps (v.3.4.0) and ggplot2 (v.3.3.5) R (v.4.0.5) packages. DRC, Democratic Republic of the Congo.

For sequences in clades 2/3, the best K resulted equal to 2 (Supplementary Figure 2). Analysis of ancestry components indicated that the 2 subpopulations define viruses sampled in Sierra Leone, Liberia, and the Ivory Coast (clade 2) and those sampled in Nigeria (clade 3). Admixture was evident for a few genomes, including the 2 rodent-derived Ghanaian samples and a virus from West Nigeria (Figure 3*A* and Figure 4). The population with lowest drift was clade 2 (Figure 3*B*).

For clade 1 genomes, the ΔK method estimated K = 4 as the most likely number of populations (Supplementary Figure 2). Evidence of admixture was detected in several genomes, and the distribution of ancestry components did not closely mirror geographic origin (Figures 3 and 4). The subpopulation with lowest F (component 1.4) was found in viruses sampled in a wide region ranging from central DRC to the Central African Republic and South Sudan. Component 1.4 was the major ancestry contribution of 2 MPXV genomes sampled from small mammals, whereas a third rodent-derived virus was admixed (Figure 4). The other, more drifted, components (1.1–1.3) were also widely distributed. In the Sankuru region, from which many genomes derive, the 4 components are all represented (Figure 4).

### Dating the Split of Monkeypox Virus Clades

Next, we aimed to date the MPXV phylogeny. To this purpose, the genome alignment was searched for evidence of recombination events using the 3SEQ algorithm, which detected 12 unique recombination events (Supplementary Figure 3). Mapping of breakpoint positions allowed the definition of a large (136 Kb) nonrecombining region, which was used for phylogenetic inference and molecular dating.

**Figure 5.**
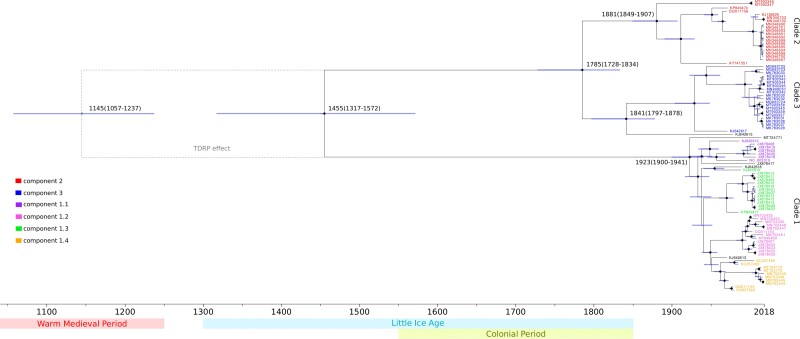
Time-scaled monkeypox virus (MPXV) phylogenetic tree. Dated maximum credibility tree estimated using the nonrecombinant region of MPXV genomes. Branch lengths represent the evolutionary time in years, and a timescale grid is shown at the tree base. Node bars indicate 95% highest posterior density intervals of node ages, and black dots indicate nodes with bootstrap support higher than 0.8. The MPXV strains are colored according to the major ancestry component identified by STRUCTURE, with black indicating admixed samples. The generally accepted time frames for the Warm Medieval Period, the Little Ice Age, and the Colonial Period are also reported [39, 40].

Reliable time estimates can only be obtained if a temporal signal is detected in the sampled sequences. Thus, we performed regression of root-to-tip genetic distances against sampling dates. Statistical significance was assessed by permutations of the sampling dates [25] and a clear temporal signal was observed (r = 0.35, P < .001) (Supplementary Figure 4). Dating of the MPXV phylogeny was thus performed using the Bayesian Evolutionary Analysis by Sampling Trees (BEAST) software. As previously suggested [1], a strict molecular clock model was applied. Estimates indicate that clade 1 separated from clades 2/3 approximately 560 years ago. This time frame corresponds to the so-called Little Ice Age (Figure 5). The split of clades 2 and 3 was instead dated approximately 1785 Common Era (CE). These 2 lineages had similar time to the most recent common ancestor (TMRCA) of 140–180 years ago (Figure 5). Conversely, clade 1 viruses were estimated to have last shared a common ancestor in 1923 (Figure 5).

Recent evidence suggested that molecular dating can be affected by the TDRP—that is, the negatively scaling of estimated rates of viral evolution with the time frame of measurement [27, 28, 41]. The TDRP has little consequence for time estimates of recent events, but it can profoundly affect estimates of events that occurred in a distant past [28]. To assess the relevance of this effect for the MPXV dating analysis, we applied a recently developed approach, namely, the PoW model [28] to convert units of branch lengths from substitutions/site to divergence time. Using this approach, time estimates for the split of clades 2 and 3 and the MRCAs of the major clades remained virtually unchanged (data not shown). However, the split of clade 1 from clades 2/3 was pushed back approximately 300 years, in a time frame within the Medieval Warm Period (Figure 5). Thus, depending on the model used, the split of MPXV populations transmitted in WA and the CB occurred ∼560–860 years ago (plus credibility intervals).

## DISCUSSION

The recent increase in the frequency of monkeypox cases in endemic regions has raised alarms that the epidemiology of the disease may be changing as the result of differences in MPXV ecology [5, 42]. The ongoing worldwide outbreak seems to have confirmed these concerns, although the events associated with the transmission of the virus outside of Africa still need to be clarified and are not within the scope of this manuscript. Indeed, it has been suggested that the virus that is spreading worldwide has different features and should be considered as a distinct entity, referred to as hMPXV1 (https://virological.org/t/urgent-need-for-a-non-discriminatory-and-non-stigmatizing-nomenclature-for-monkeypox-virus/853). Our aim was thus to study the genetic diversity and separation time frame of MPXV in endemic areas (ie, where the virus has spent the overwhelming majority of its evolutionary history), because previous phylogenetic analyses included a limited number of viral genomes or restricted analyses to specific geographic regions [12–14].

Our results do not provide unequivocal information about the location where MPXV first emerged. However, the low drift of clades 2/3 is consistent with an origin in West Africa. Viral expansion in the Congo Basin was possibly accompanied by a bottleneck or founder effect. Molecular dating indicates that clade 1 separated from clades 2/3 in a period ranging from the Medieval Warm Period to the Little Ice Age. Analysis of lake sediments in Gabon showed that the Medieval Warm Period was characterized by fluctuations of wet and dry conditions, whereas a dry climate was prevalent during the subsequent Little Ice Age [43]. Such hydrological changes affected the dynamics of the tropical rainforests, which expanded during high rainfall periods and contracted when precipitation decreased [43]. These variable conditions might have opened ecologically favorable corridors for the MPXV reservoir(s) to migrate into the Congo Basin region. The subsequent shrinking of forest area might have resulted in geographical isolation of both the reservoir and the virus. In line with this possibility, the geographic range of Thomas’s rope squirrel (*Funisciurus anerythrus*), considered a likely reservoir of MPXV [10, 11], includes both West and Central Africa (Figure 4). However, the 2 squirrel populations seem to be isolated, with no continuity in their geographic range (Figure 4).

It is unfortunate that large uncertainties still exist about the host range of MPXV in natural settings. The virus has been detected in several small mammals, including the above-mentioned rope squirrels, pouched rats, African dormice, and shrews [6, 10, 11, 44]. Experimental or accidental infection has also indicated that the virus has a relatively wide host range [6]. Most of the genomes we analyzed derived from human infections. However, 3 viruses in clade 1 were obtained from 2 rope squirrels (Lunda rope squirrel and Thomas’s rope squirrel) and a naked-tail shrew (*Crocidura littoralis*). All of these viruses contain a considerable fraction of the clade 1 component showing the lowest drift, which is consistent with the role of squirrels as the original entry vectors of MPXV in the Congo Basin. A recent analysis of museum specimens from Central Africa detected MPXV-genomic DNA in specimens from different species of African rope squirrels (*Funisciurus* spp), the highest prevalence being observed for *F anerythrus* and *Funisciurus congicus* [11]. The latter has a host range restricted to Central Africa (https://www.iucnredlist.org/). Likewise naked-tail shrews have not been reported in West Africa (https://www.iucnredlist.org/). It is thus possible that the separation of clades 1 and 2/3 was not only determined by the ecological opportunity of the natural hosts to colonize a new geographic area but also by host shifts that allowed establishment of novel reservoirs. In this respect, it is also worth noting that, in contrast to clades 2/3, the genetic diversity of clade 1 is not geographically structured. A possible explanation for this observation is that the distinct subpopulations we detected represent viral circulation in distinct host species. Alternatively, the main viral reservoir(s) might be capable of long-range movements.

Whereas additional sampling, possibly from the wildlife, will help to disentangle these possibilities, the deep split of clades 1 and 2/3 as well as the shallow TMRCA of the former indicate a considerable amount of unsampled viral diversity or extinction of early lineages. Viral lineage extinction is thought to be a common phenomenon and it was also documented for VARV [45–47]. In the case of MPXV, the variable climate conditions during the Little Ice Age or encroachment of forest habitats during the colonial period might have affected reservoir populations and caused viral lineage extinction.

Compared to clade 1, clades 2/3 suffered less drift. In addition, as in the case of clade 1, the long branch from the root suggests unsampled diversity or lineage extinction, whose causes might be similar to those hypothesized above for clade 1 viruses. Clades 2/3 show strong geographic structuring of genetic diversity, with the Dahomey Gap (which interrupts the rainforest with a savanna region) possibly acting as a geographic barrier. A minority of admixed genomes were sampled at the boundaries of the regions dominated by the clade 2 and clade 3 components. Two of these admixed viruses derive from Ghana and were sequenced from animals: a dormouse and a pouched rat. These are also considered possible reservoirs for MPXV, and the existence of distinct hosts in West Africa is also supported by the observation that the geographic range of *F anerythrus* does not extend West of Nigeria. However, the role of (1) these putative reservoirs in the epidemiology of monkeypox and (2) the involvement of additional unidentified hosts remains largely unknown. In recent studies, analysis of MPXV infection in chimpanzee communities indicated that disease outbreaks were not caused by increased rodent consumption. Moreover, viable virus could be detected in flies [1]. Although our data cannot address these open issues, they provide information about the demographic history and evolution of MPXV and suggest that viral ecology differs in West Africa and in the Congo Basin.

## CONCLUSIONS

Our study clearly has limitations. First, the available number of MPXV sequences is small and their geographic distribution quite uneven, both in West Africa and in the Congo Basin. This clearly limits spatial resolution and possibly introduces biases. Biases are also likely to be caused by the fact that the overwhelming majority of sequences were derived from human infections. On one hand, this prevents a comprehensive assessment of MPXV genetic diversity; on the other hand, sampled genomes might preferentially derive from hosts that are frequently hunted or that come into contact with humans (ie, commensal or pericommensal species). These effects might be relevant, because all the approaches we applied are based on the assumption that the analyzed sequences represent an unbiased sample of the viral population. For STRUCTURE analysis, using priors that account or do not account for unequal sampling did not change the results. Although this suggests that sampling does not largely affect our conclusion, it is not demonstration thereof. In addition, unequal sampling is expected to affect dating analysis and summary statistics. Biased sampling in molecular dating is a well known problem [48] with limited solutions, especially when the availability of genetic data is scant (as it is here). Thus, additional sequencing of MPXV genomes will be instrumental to provide insight into the evolution of the viral populations and to validate the robustness of our findings. In particular, large-scale, field studies in wild animals will be particularly valuable to increase genomic surveillance and decrease sampling biases. This will be relevant for both a better understanding of monkeypox epidemiology in the endemic region and to gain insight into the events that originated and are sustaining the current multicountry outbreak.

## Supplementary Data

Supplementary materials are available at *The Journal of Infectious Diseases* online. Supplementary materials consist of data provided by the authors that are published to benefit the reader. The posted materials are not copyedited. The contents of all supplementary data are the sole responsibility of the authors. Questions or messages regarding errors should be addressed to the author.

## Notes

***Financial support*.** This work was funded by the Italian Ministry of Health (“Ricerca Corrente 2022”; to M. S.).

## Supplementary Material

jiac298_Supplementary_Data
