# Supplementary material for: Geographic Structuring and Divergence Time Frame of Monkeypox Virus in the Endemic Region
Source: J Infect Dis. 2022 Jul 14;227(6):742–51. doi: 10.1093/infdis/jiac298 (PMC10044091; doi:10.1093/infdis/jiac298)

Supplementary Methods

Sequences, alignments, network, and nucleotide diversity

Complete MPXV genomes were retrieved from the NCBI virus database (https://www.ncbi.nlm.nih.gov/labs/virus/vssi/#/). Viral sequences generated by artificial recombination or other approaches were not included. Identical genome sequences were removed and a final set of 90 viral strains was obtained (Supplementary Table 1). MAFFT (v.7.475) with default parameters was used to generate the whole genome alignment [1]. Unrooted phylogenetic networks were generated using SplitsTree4 (v4.16.2) [2] with HKY85 distances, all polymorphic sites, and without gap sites.

Linkage disequilibrium and population structure

Population structure analysis was performed by considering biallelic parsimony-informative (PI) sites. In particular, we selected biallelic sites, each with a minimum count of two (i.e., non-singletons), for those genomic positions where at least 50% of sequences had non-missing information. Gaps and all nonstandard nucleotide bases were considered as missing values. This generated a list of 1547, 777, and 216 variants for the whole dataset, clades 2/3, and clade 1, respectively.

To evaluate linkage disequilibrium (LD) we used the LIAN software (v.3.7) [3], that tests for independent assortment by computing the number of loci at which each pair of haplotypes differs. Significance was assessed by Monte Carlo simulations (1,000 iterations). The interpretation of LD was associated to the standardized index of association (I_AS), displayed by LIAN analysis (i.e. zero means linkage equilibrium).

The STRUCTURE (v.2.3.4) [4] software was applied to evaluate viral population structure. This tool divides the whole population into K subpopulations characterized by a set of allele frequencies

at each locus [4] without any *a priori* information. We first estimated the λ (allele frequency spectrum) parameter by running STRUCTURE with K=1, as suggested (the parameter file is provided at https://zenodo.org/record/6809635#.YsfYr9JBzs0), and we then applied the linkage model with correlated allele frequencies [5] for K from 1 to 12. This model extends the admixture model to (weakly) linked loci and it has good power to detect subtle population structure (the parameter file is provided at https://zenodo.org/record/6809635#.YsfYr9JBzs0)[5]. For each K, ten runs were performed with a MCMC chain length of 500,000 iterations and 50,000 burn-in and map distances were set equal to PI site physical distances. The optimal K was evaluated using the HARVESTER tool [6], according to Evanno's method[7]. The CLUMPAK [8] software was used to combine replicate runs from the same K and to generate the Q value matrix.

Molecular dating and PoW model

For of root-to-tip regression, we first generated a phylogenetic tree for the nonrecombinant region using RAxML (v.8.2.12) [9] by running a rapid bootstrapping and subsequent ML search, with cowpox virus as an outgroup. A method that minimizes the residual mean squares of the models was applied [10]. Statistical significance was calculated by performing 1,000 permutations of the tip dates [10].

By taking advantage of the heterochronous dataset and the presence of a temporal signal, we performed a time estimate phylogenetic reconstruction using a Bayesian approach implemented in the Bayesian Evolutionary Analysis by Sampling Trees (BEAST, v.1.10.4) software [11]. Analyses were performed using a Bayesian MCMC method with a strict clock model and a constant population size tree prior, as previously suggested [12, 13]. A HKY85 substitution model with a gamma-distributed rate variation among sites was selected [12, 13]. We performed two different runs, a hundred million iterations each, and sampled every 10,000 steps after a 10% burn-in (BEAST input file is provided at https://zenodo.org/record/6809635#.YsfYr9JBzs0). Runs were checked with Tracer (v.1.7.1) [14] for convergence and for having ESS (effective sampling size)

values >200 and then combined with logCombiner [11]. Finally, we built a maximum clade credibility tree using TreeAnnotator {[11], which was visualized with FigTree (http://tree.bio.ed.ac.uk/).

For the PoW model, we constructed 10,000 ultrametric distance trees with BEAST using the HKY85 substitution model and subsampled 500 random distance trees. We then converted each of them to a time tree using the median substitution rate inferred from 500 random samples from the posterior rate distribution calculated in the previous dating analysis and by applying the proposed model [15]. Finally, using TreeAnnotator, we generated a TRDP-aware consensus tree.

Supplementary References

1. Katoh K, Standley DM. MAFFT multiple sequence alignment software version 7: improvements in performance and usability. Mol Biol Evol **2013**;30:772-80.

2. Huson DH, Bryant D. Application of phylogenetic networks in evolutionary studies. Mol Biol Evol **2006**;23:254-67.

3. Haubold B, Hudson RR. LIAN 3.0: detecting linkage disequilibrium in multilocus data. Linkage Analysis. Bioinformatics **2000**;16:847-8.

4. Pritchard JK, Stephens M, Donnelly P. Inference of population structure using multilocus genotype data. Genetics **2000**;155:945-59.

5. Falush D, Stephens M, Pritchard JK. Inference of population structure using multilocus genotype data: linked loci and correlated allele frequencies. Genetics **2003**;164:1567-87.

6. Earl DA, vonHoldt BM. STRUCTURE HARVESTER: a website and program for visualizing STRUCTURE output and implementing the Evanno method. Cons Genet Res **2012**;4:359-61.

7. Evanno G, Regnaut S, Goudet J. Detecting the number of clusters of individuals using the software STRUCTURE: a simulation study. Mol Ecol **2005**;14:2611-20.

8. Kopelman NM, Mayzel J, Jakobsson M, Rosenberg NA, Mayrose I. Clumpak: a program for identifying clustering modes and packaging population structure inferences across K. Mol Ecol Resour **2015**;15:1179-91.

9. Stamatakis A. RAxML version 8: a tool for phylogenetic analysis and post-analysis of large phylogenies. Bioinformatics **2014**;30:1312-3.

10. Murray GG, Wang F, Harrison EM, et al. The effect of genetic structure on molecular dating and tests for temporal signal. Methods Ecol Evol **2016**;7:80-9.

11. Suchard MA, Lemey P, Baele G, Ayres DL, Drummond AJ, Rambaut A. Bayesian phylogenetic and phylodynamic data integration using BEAST 1.10. Virus Evol **2018**;4:vey016.

12. Patrono LV, Pléh K, Samuni L, et al. Monkeypox virus emergence in wild chimpanzees reveals distinct clinical outcomes and viral diversity. Nat Microbiol **2020**;5:955-65.

13. Berthet N, Descorps-Declère S, Besombes C, et al. Genomic history of human monkey pox infections in the Central African Republic between 2001 and 2018. Sci Rep **2021**;11:13085,021-92315-8.

14. Rambaut A, Drummond AJ, Xie D, Baele G, Suchard MA. Posterior Summarization in Bayesian Phylogenetics Using Tracer 1.7. Syst Biol **2018**;67:901-4.

15. Ghafari M, Simmonds P, Pybus OG, Katzourakis A. A mechanistic evolutionary model explains the time-dependent pattern of substitution rates in viruses. Curr Biol **2021**;31:4689,4696.e5.

Supplementary Table 1. List of analyzed strains.

Accession	Strain	Country	Region/prefecture	Isolation year	Host	Pubmed ID
AY741551	Sierra Leone	Sierra Leone	Sierra Leone	1970	*NA*	16023693
DQ011156	Liberia_1970_184	Liberia	Liberia	1970	*Homo sapiens*	16186219
KJ136820	Ivory Coast 2012	Ivory coast	Ivory coast Tai national park	2012	*wild monkey*	24857667
KP849470	Cote d'Ivoire_1971	Ivory coast	Ivory coast	1971	*NA*	25912718
MN346690	MPXV_TNP_2017_North_Bic	Ivory coast	Ivory coast Tai national park	2017	*Pan troglodytes verus*	32341480
MN346691	MPXV_TNP_2017_North_Environmental	Ivory coast	Ivory coast Tai national park	2017	*environment*	32341480
MN346692	MPXV_TNP_2017_North_Mama	Ivory coast	Ivory coast Tai national park	2017	*Pan troglodytes verus*	32341480
MN346693	MPXV_TNP_2017_North_Ponan	Ivory coast	Ivory coast Tai national park	2017	*Pan troglodytes verus*	32341480
MN346694	MPXV_TNP_2017_North_Saro	Ivory coast	Ivory coast Tai national park	2017	*Pan troglodytes verus*	32341480
MN346695	MPXV_TNP_2017_North_Sidonie	Ivory coast	Ivory coast Tai national park	2017	*Pan troglodytes verus*	32341480
MN346696	MPXV_TNP_2017_North_Surprise_1	Ivory coast	Ivory coast Tai national park	2017	*Pan troglodytes verus*	32341480
MN346697	MPXV_TNP_2017_North_Surprise_2	Ivory coast	Ivory coast Tai national park	2017	*Pan troglodytes verus*	32341480
MN346698	MPXV_TNP_2017_South_Pushkin	Ivory coast	Ivory coast Tai national park	2017	*Pan troglodytes verus*	32341480
MN346699	MPXV_TNP_2017_South_Ravel_1	Ivory coast	Ivory coast Tai national park	2017	*Pan troglodytes verus*	32341480
MN346700	MPXV_TNP_2017_South_Ravel_2	Ivory coast	Ivory coast Tai national park	2017	*Pan troglodytes verus*	32341480
MN346701	MPXV_TNP_2017_South_Woodstock	Ivory coast	Ivory coast Tai national park	2017	*Pan troglodytes verus*	32341480
MN346702	MPXV_TNP_2018_East_Paddy	Ivory coast	Ivory coast Tai national park	2018	*Pan troglodytes verus*	32341480
MN346703	MPXV_TNP_2018_East_Placali	Ivory coast	Ivory coast Tai national park	2018	*Pan troglodytes verus*	32341480
MT903346	MPXV-USA2003_099_Gambian_Rat	Ghana	Ghana	2003	*Cricetomys gambianus*	32880628
MT903347	MPXV-USA2003_099_Dormouse	Ghana	Ghana	2003	*dormouse*	32880628
KJ642615	W-Nigeria	Nigeria	Nigeria,Omifunfun	1978	*NA*	25912718
MT903341	MPXV-M5320_M15_Bayelsa	Nigeria	Nigeria,Bayelsa	2018	*Homo sapiens*	32880628

MK783031	3020	Nigeria	Nigeria,Rivers State	2017	*Homo sapiens*	31285143
MT903338	MPXV-M2957_Lagos	Nigeria	Nigeria,Lagos	2017	*Homo sapiens*	32880628
MT903342	MPXV-Singapore	Nigeria	Nigeria,Ebonyi	2018	*Homo sapiens*	32880628
KJ642617	Nigeria-SE-1971	Nigeria	Nigeria,Ihie	1971	*NA*	25912718
MK783027	3018	Nigeria	Nigeria,Rivers State	2017	*Homo sapiens*	31285143
MK783028	3019	Nigeria	Nigeria,Rivers State	2017	*Homo sapiens*	31285143
MK783029	3029	Nigeria	Nigeria,Rivers State	2017	*Homo sapiens*	31285143
MK783030	3025	Nigeria	Nigeria,Rivers State	2017	*Homo sapiens*	31285143
MK783032	3030	Nigeria	Nigeria,Rivers State	2017	*Homo sapiens*	31285143
MK783033	2920	Nigeria	Nigeria,Rivers State	2017	*Homo sapiens*	31285143
MN648051	Israel_2018	Nigeria	Nigeria,Rivers State	2018	*Homo sapiens*	32139560
MT903337	MPXV-M2940_FCT	Nigeria	Nigeria,Federal Capital Territory	2017	*Homo sapiens*	32880628
MT903339	MPXV-M3021_Delta	Nigeria	Nigeria,Delta	2017	*Homo sapiens*	32880628
MT903340	MPXV-M5312_HM12_Rivers	Nigeria	Nigeria,Rivers State	2017	*Homo sapiens*	32880628
MT903343	MPXV-UK_P1	Nigeria	Nigeria	2018	*Homo sapiens*	32880628
MT903344	MPXV-UK_P2	Nigeria	Nigeria	2018	*Homo sapiens*	32880628
MT903345	MPXV-UK_P3	Nigeria	Nigeria	2018	*Homo sapiens*	32880628
MG693723	MPXV_Nig_2017_297957	Nigeria	Nigeria,Yenagoa	2017	*Homo sapiens*	29361427
MG693724	MPXV_Nig_2017_298464	Nigeria	Nigeria,Yenagoa	2017	*Homo sapiens*	29361427
MG693725	MPX_Nig_2017_298481	Nigeria	Nigeria,Yenagoa	2017	*Homo sapiens*	29361427
KC257459	Sudan 2005_01	South Sudan	South Sudan	2005	*Homo sapiens*	23347770
MT724772	Funisciurus_anerythrus_2014_DRC	DRC	Democratic Republic of the Congo,Kisangani	2014	*Funisciurus anerythrus*	https://www.researchsquare.com/article/rs-414280/v1
MT724771	MPXV_DRC_2014_Funisciurus_bayonii_Y081	DRC	Democratic Republic of the Congo,Kisangani	2014	*Funisciurus bayonii*	https://www.researchsquare.com/article/rs-414280/v1
JX878417	DRC 07-0104	DRC (Sankuru)	Democratic Republic of the Congo,Bena-Dibele Health Zone	2006	*Homo sapiens*	24457084
NC_003310	Zaire-96-I-16	DRC	Democratic Republic of the Congo,Akungula	1996	*NA*	11734207
DQ011154	Congo_2003_358	Congo Republic	Congo Republic,Impfondo	2003	*Homo sapiens*	16186219
DQ011155	Zaire_1979-005	DRC (Sankuru)	Democratic Republic of the Congo,Mindembo	1978	*Homo sapiens*	16186219
HQ857562	V79-I-005	DRC (Sankuru)	Democratic Republic of the Congo,Mindembo	1979	*Homo sapiens*	21752919
JX878407	DRC 06-0950	DRC (Sankuru)	Democratic Republic of the Congo,Kole Health Zone	2006	*Homo sapiens*	24457084
JX878408	DRC 06-0970	DRC (Sankuru)	Democratic Republic of the Congo,Katako Kombe Health Zone	2006	*Homo sapiens*	24457084

JX878409	DRC 06-0999	DRC (Sankuru)	Democratic Republic of the Congo,Vangakete Health Zone	2006	*Homo sapiens*	24457084
JX878410	DRC 06-1070	DRC (Sankuru)	Democratic Republic of the Congo,Vangakete Health Zone	2006	*Homo sapiens*	24457084
JX878411	DRC 06-1075	DRC (Sankuru)	Democratic Republic of the Congo,Vangakete Health Zone	2006	*Homo sapiens*	24457084
JX878412	DRC 06-1076	DRC (Sankuru)	Democratic Republic of the Congo,Vangakete Health Zone	2006	*Homo sapiens*	24457084
JX878413	DRC 07-0045	DRC (Sankuru)	Democratic Republic of the Congo,Lomela Health Zone	2006	*Homo sapiens*	24457084
JX878414	DRC 07-0046	DRC (Sankuru)	Democratic Republic of the Congo,Lomela Health Zone	2006	*Homo sapiens*	24457084
JX878415	DRC 07-0092	DRC (Sankuru)	Democratic Republic of the Congo,Lomela Health Zone	2006	*Homo sapiens*	24457084
JX878416	DRC 07-0093	DRC (Sankuru)	Democratic Republic of the Congo,Lomela Health Zone	2006	*Homo sapiens*	24457084
JX878418	DRC 07-0120	DRC (Sankuru)	Democratic Republic of the Congo,Djalo-Ndjeka Health Zone	2007	*Homo sapiens*	24457084
JX878419	DRC 07-0275	DRC (Sankuru)	Democratic Republic of the Congo,Djalo-Ndjeka Health Zone	2007	*Homo sapiens*	24457084
JX878420	DRC 07-0283	DRC (Sankuru)	Democratic Republic of the Congo,Djalo-Ndjeka Health Zone	2007	*Homo sapiens*	24457084
JX878421	DRC 07-0286	DRC (Sankuru)	Democratic Republic of the Congo,Lomela Health Zone	2007	*Homo sapiens*	24457084
JX878422	DRC 07-0287	DRC (Sankuru)	Democratic Republic of the Congo,Djalo-Ndjeka Health Zone	2007	*Homo sapiens*	24457084
JX878423	DRC 07-0337	DRC (Sankuru)	Democratic Republic of the Congo,Kole Health Zone	2007	*Homo sapiens*	24457084
JX878424	DRC 07-0338	DRC (Sankuru)	Democratic Republic of the Congo,Kole Health Zone	2007	*Homo sapiens*	24457084
JX878425	DRC 07-0354	DRC (Sankuru)	Democratic Republic of the Congo,Lomela Health Zone	2007	*Homo sapiens*	24457084
JX878426	DRC 07-0450	DRC (Sankuru)	Democratic Republic of the Congo,Kole Health Zone	2007	*Homo sapiens*	24457084
JX878427	DRC 07-0480	DRC (Sankuru)	Democratic Republic of the Congo,Lomela Health Zone	2007	*Homo sapiens*	24457084
JX878428	DRC 07-0514	DRC (Sankuru)	Democratic Republic of the Congo,Lomela Health Zone	2007	*Homo sapiens*	24457084
JX878429	DRC 07-0662	DRC (Sankuru)	Democratic Republic of the Congo,Lomela Health Zone	2007	*Homo sapiens*	24457084
KC257460	DRC Yandongi 1985	DRC	Democratic Republic of the Congo,Yandongi	1985	*Homo sapiens*	23347770

KJ642612	Ikubi	DRC	Democratic Republic of the Congo,Ikubi	1986	*NA*	25912718
KJ642613	Congo_8	DRC	Democratic Republic of the Congo,Bokenda	1970	*NA*	25912718
KJ642618	Cameroon-1990	Cameroon	Cameroon	1990	*NA*	25912718
KJ642619	Gabon-1988	Gabon	Gabon	1988	*NA*	25912718
KP849469	Boende_DRC_2008	DRC	Democratic Republic of the Congo,Boende	2008	*NA*	25912718
KP849471	Yambuku_DRC_1985	DRC	Democratic Republic of the Congo,Yambuku	1985	*NA*	25912718
MN702444	A5_contig_SPADES	CAR	Central African Republic,Bao	2017	*Homo sapiens*	34158533
MN702445	A4_contig_SPADES	CAR	Central African Republic,Bao	2017	*Homo sapiens*	34158533
MN702446	38c_contig_SPADES	CAR	Central African Republic,Rafai	2018	*Homo sapiens*	34158533
MN702447	18_contig_SPADES	CAR	Central African Republic,Rafai	2018	*Homo sapiens*	34158533
MN702448	015c_contig_SPADES	CAR	Central African Republic,Ippy	2018	*Homo sapiens*	34158533
MN702449	B2_contig_SPADES	CAR	Central African Republic,Bangassou	2016	*Homo sapiens*	34158533
MN702450	B1_contig_SPADES	CAR	Central African Republic,Bakouma	2016	*Homo sapiens*	34158533
MN702451	A6_contig_SPADES	CAR	Central African Republic,Mbaiki	2017	*Homo sapiens*	34158533
MN702452	A2_contig_SPADES	CAR	Central African Republic,Mongoumba	2010	*Homo sapiens*	34158533
MN702453	A1_contig_SPADES	CAR	Central African Republic,Bangassou	2001	*Homo sapiens*	34158533
MT724770	MPXV_DRC_2014_Crocidura_littoralis_COB771	DRC	Democratic Republic of the Congo,Kisangani	2014	*Crocidura littoralis*	https://www.researchsquare.com/article/rs-414280/v1

Supplementary Figure 1. Sliding window analysis of Tajima's D. A genome wide sliding-window analysis (window length=5000 nucleotides, window step=1000 nucleotide) is plotted for clade 1 (blue line) and for clade 2/3 (red line) genomes. Calculations were performed using the POP-GENOME R package.

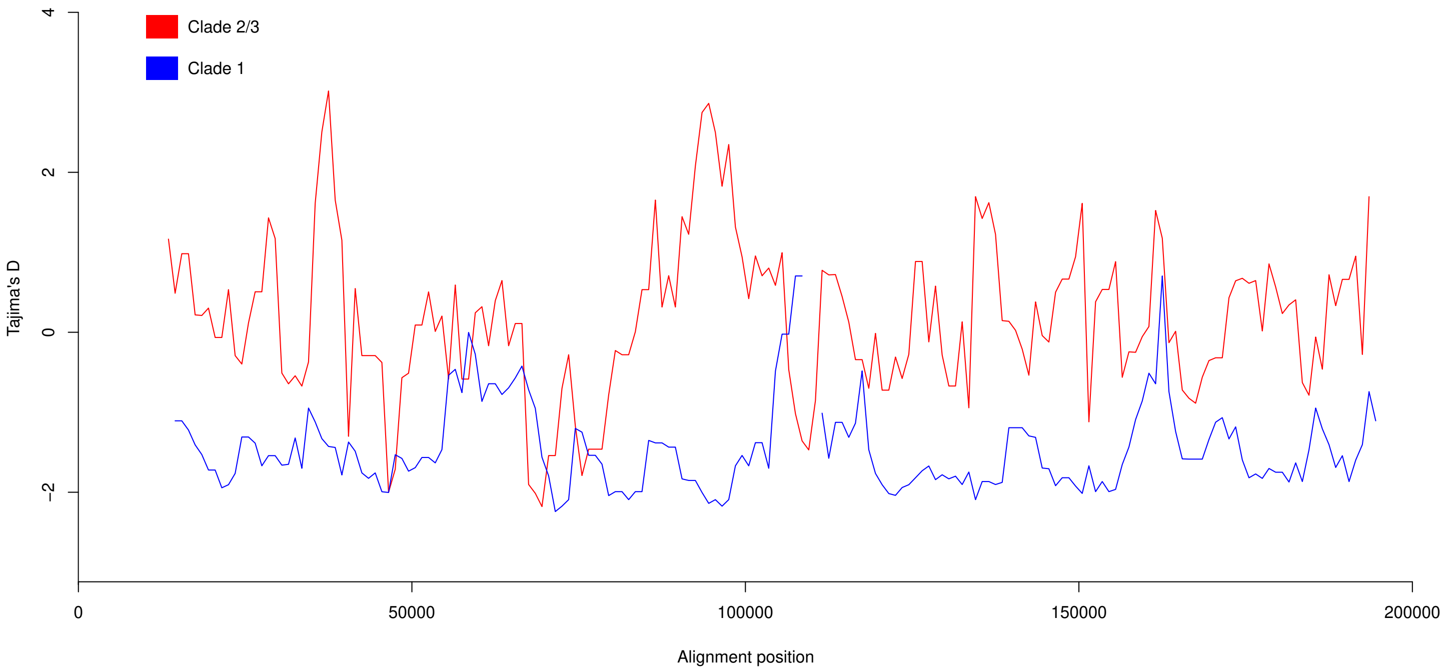

Supplementary Figure 2. Selection of the optimal K. Analysis of optimal K for STRUCTURE analysis for (A) the whole dataset, (B) the WA clade, and (C) the CB clade. ΔK is calculated as ΔK =mean(|L''(K)|) / sd(L(K)). The peak of each distribution is the optimal K used in STRUCTURE analysis.

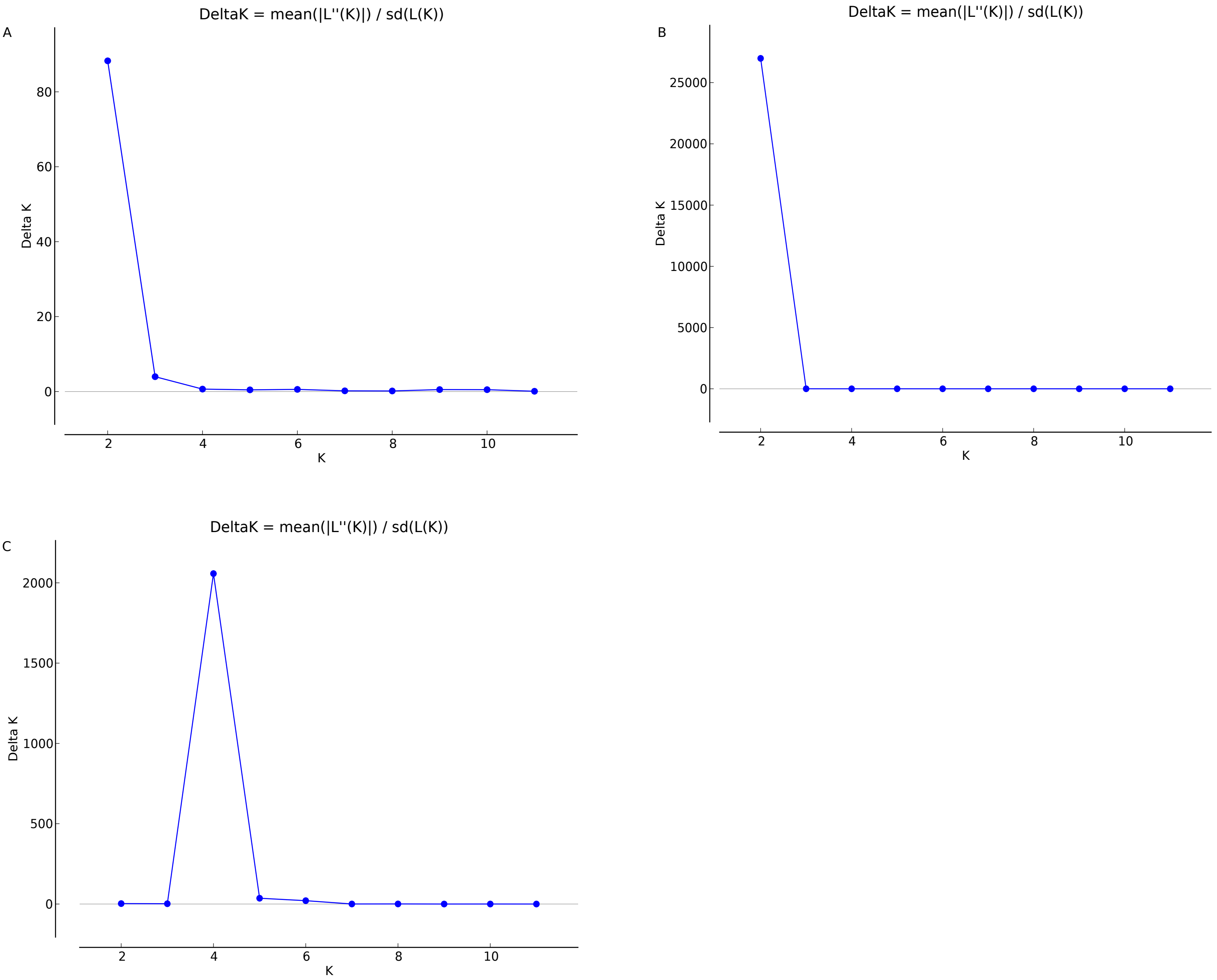

Supplementary Figure 3. MPXV recombination events. Unique recombination events in 90 MPXV genomes. Each event is shown as a line with dots representing the start and the end. The location ofselected nonrecombinant region used in the analyses is shown in red. Positions refer to the whole genome alignment (corresponding positions in the NCBI Reference Sequence NC_003310: 35,195-167,795).

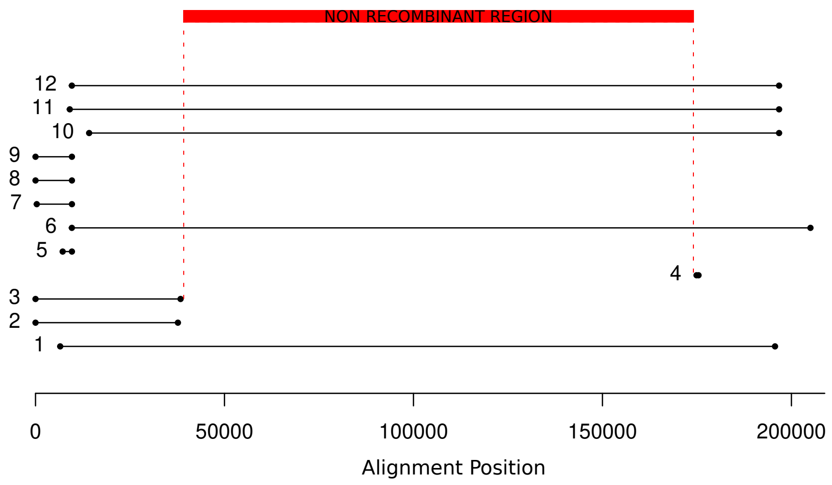

Supplementary Figure 4. Temporal signal. Plot of the root-to-tip distance as a function of sampling years. Each point corresponds to a viral sequence and the gray line is the linear regression calculated using a method that minimizes the residual mean squares. The r coefficient and the corresponding pvalue (generated after 1000 permutations) are also shown.

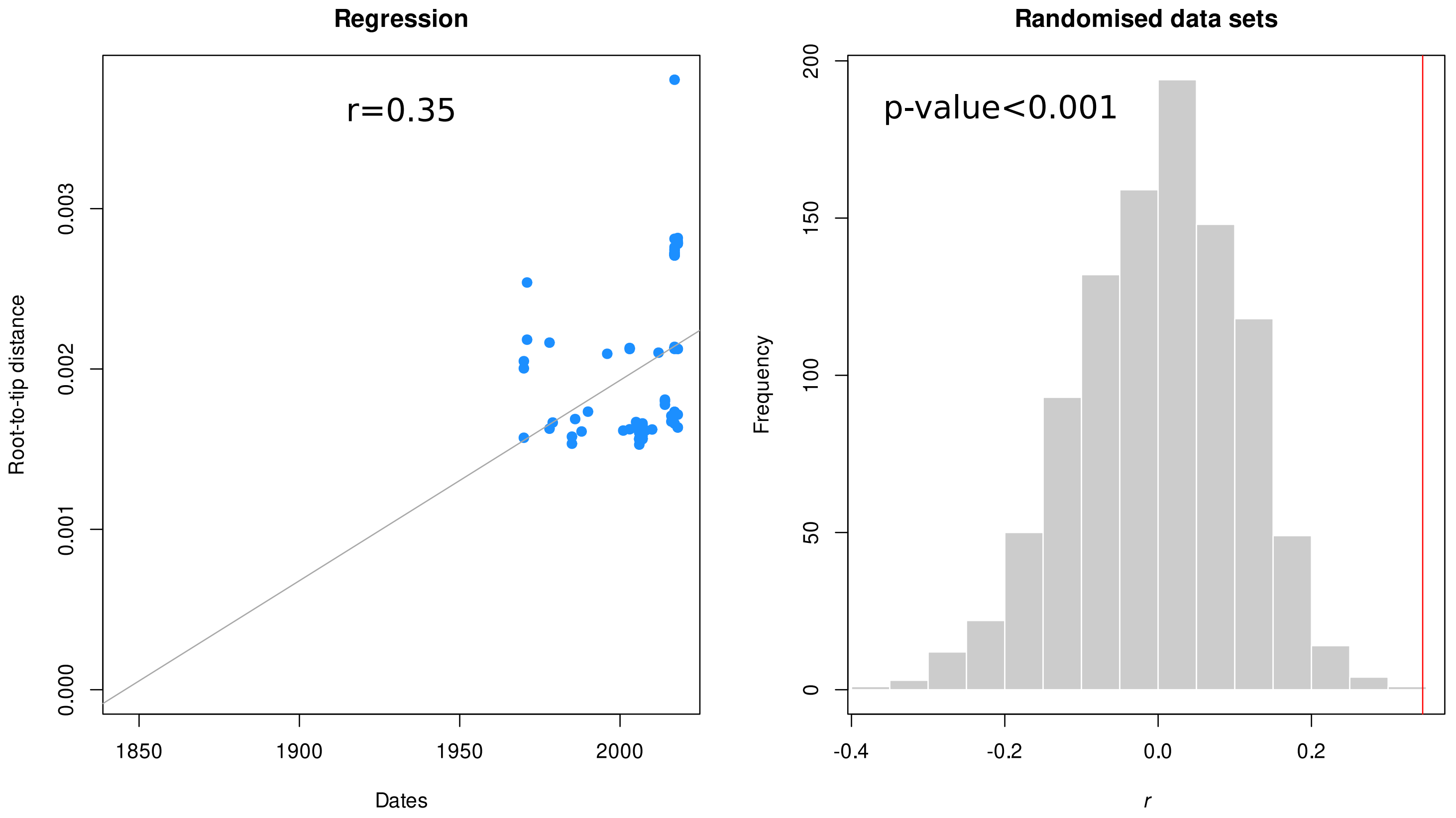

Supplement: jiac298_Supplementary_Data [file jiac298_supplementary_data.pdf]
